# CLEVER maternity care: A before-and-after study of women’s experience of childbirth in Tshwane, South Africa

**DOI:** 10.4102/phcfm.v12i1.2560

**Published:** 2020-10-15

**Authors:** Sarie J. Oosthuizen, Anne-Marie Bergh, Jackie Grimbeek, Robert C. Pattinson

**Affiliations:** 1 Tshwane District Health and Department of Family Medicine, University of Pretoria, Pretoria, South Africa; 2 Research Centre for Maternal, Fetal, Newborn and Child Health Care Strategies, Faculty of Health Sciences, University of Pretoria, Pretoria, South Africa; 3 UP/SAMRC Unit for Maternal and Infant Health Care Strategies, Faculty of Health Sciences, University of Pretoria, Pretoria, South Africa

**Keywords:** respectful maternal care, obstetric care, childbirth, quality improvement

## Abstract

**Background:**

Birthing care matters to women and some women experience mistreatment during childbirth.

**Aim:**

To determine the effect the ‘CLEVER Maternity Care’ package, a multi-faceted intervention to improve respectful, quality obstetric care.

**Setting:**

Ten midwife-led obstetric units in Tshwane health district, South Africa; five intervention and five control units.

**Methods:**

We conducted an anonymous baseline and end-line survey to measure the change in women’s perceptions and experiences of childbirth care after the implementation of the CLEVER package. A convenience sample of women returning for a postnatal follow-up visit was obtained at baseline (*n* = 653) and after implementation of CLEVER (*n* = 679).

**Results:**

Six survey items were selected as proxies for respectful clinical care. There was no significant change in proportions of responses regarding one question, and with regard to patients receiving attention within 15 min of arrival, both the intervention and control group units showed a significant increase in positive responses (odds ratios of 8.4 and 6.1, respectively, and *p* values of 0.0001 and 0.0007). For the remaining four items (asking permission before doing an examination, positive communication, respectful treatment and overall satisfaction), only the intervention group showed a significant positive change (odds ratios ranging from 2.4 to 4.3; *p* ≤ 0.0018), with no significant change for the control group (odds ratios between 1.0 and 1.8; *p* ≥ 0.0736).

**Conclusion:**

After the implementation of CLEVER Maternity Care, women reported a more positive experience of childbirth. The CLEVER intervention is a potential strategy for addressing respectful, quality obstetric care that warrants further investigation.

## Background

Health services need to achieve the optimal balance between what health systems can provide in low- and middle-income countries (LMICs) and what women expect during birthing.^1,2^ There is a shift towards respectful, high-quality obstetric care^3^ as well as a renewed focus by the World Health Organization (WHO) on improving maternal and neonatal mortality and morbidity in LMICs.^4^ Birthing care during labour should be a supportive interaction between a woman and the healthcare providers, with attention to respectful care and meeting the sociocultural, emotional and psychological expectations and needs of the woman.^5^ Women’s experience of mistreatment during childbirth could contribute to poor health and issues with relationships.^6^

Skilled birth attendants who provide quality care during labour and childbirth and behave respectfully could improve women’s satisfaction with childbirth^3^ and also ensure maternal and newborn well-being.^7^ Strategies for improving respectful maternity care need to follow a collaborative improvement approach^8,9^ that incorporates health-systems barriers and systems thinking into pathways of better-sustained care.^10,11,12,13^

Bowser and Hill’s landscape analysis of abuse in facility-based childbirth^14^ has been followed by other studies highlighting the mistreatment of birthing women.^15,16,17,18^ Standards for the quality of maternal and newborn care are well documented.^19^ Although health professionals subscribe to quality birthing care and accept that a woman is entitled to human rights and deserves respectful treatment,^20^ some fall short in the areas of effective communication and shared decision-making, respectful and dignified care and emotional support during labour.^21^

Many studies in LMICs have highlighted the barriers and the actions required to achieve respectful birthing care.^22,23,24,25^ Quality improvement of care in labour wards should not neglect the professional barriers faced by birth attendants.^26^ Poor functioning of the broader health system, as described in the WHO’s building blocks,^27^ can lead to omissions in birthing care that negatively influence the experience for birthing women as well as health professionals.

Studies from South Africa and other LMICs attempted to understand why mistreatment was so prevalent during facility births.^15,16,18,28,29^ A mixed-methods systematic review highlights the following themes of discrimination based on sociodemographic characteristics such as age, ethnicity, race and religion; failure to meet professional standards of care; poor rapport and ineffective communication between women and their care providers; lack of supportive care; and loss of autonomy.^30^ Health-systems constraints include lack of resources, absence of policies and a facility culture aimed at protecting women from mistreatment.^30^

CLEVER Maternity Care^31^ is an intervention package that focuses on achieving high-quality respectful obstetric care in midwife-led obstetric units (MOUs). The acronym CLEVER stands for **C**linical care and obstetric triage; **L**abour ward management to resolve the withholding of care; **E**liminate barriers to meet basic human needs; **V**erify care with monitoring, evaluation and feedback to reach reflective practice; **E**mergency obstetric simulation training (EOST) to create autopilot sequences during emergencies; and **R**espectful care to improve birthing women’s experiences.^31^

The effects of the pilot implementation of the CLEVER package in the Tshwane health district, South Africa, were measured in terms of perinatal morbidity and mortality as proxies for quality obstetric care and in terms of women’s experience of childbirth. Results on the significant improvements in the rates of fresh stillbirths, birth asphyxia and meconium aspiration have been reported elsewhere.^31^ This paper reports the changes in women’s experiences of childbirth before and after the implementation of the CLEVER package in the Tshwane district. The research question was as follows: which of the experiences of women delivering in intervention MOUs changed significantly from baseline to end-line when compared with those of women delivering in control MOUs?

## Methods

### Study design

The study reported in this paper was part of the pilot implementation of the broader CLEVER intervention. This study entailed a survey of women’s experiences of childbirth and early postnatal care before the start of the intervention, which was repeated after the end of the intervention.

### Study setting

The study was conducted in the Tshwane health district. The district had about 50 000 deliveries per annum, of which 18% were conducted in its MOUs. Midwife-led obstetric units are primary healthcare units that provide 24-h maternity services and are attached to community health centres. All 10 MOUs in the district were included in the study. There were five non-intervention MOUs. Five MOUs were purposively selected to receive the CLEVER intervention. The latter five were the more underserved facilities located in one geographical area under the same area manager. This minimised intervention spillover to the control MOUs.

### Intervention

The CLEVER Maternity Care package was implemented in three phases. The first phase consisted of a period of creating awareness, soliciting participation from MOUs and carrying out activities aimed at strengthening health systems. Creating awareness included giving feedback of women’s experiences of their birthing treatment in MOUs using a baseline survey.^32^ Experience was used as an umbrella term that also encompassed perceptions and satisfaction. The second phase addressed a core group of activities aimed at behavioural change. These consisted of an intensive 3-month engagement with each intervention MOU, followed by a further 6 months of follow-up support to improve and sustain respectful and safe clinical care practices. The same survey of women’s experiences was repeated in the third phase of the intervention as part of the end-line measurement of the effects of working CLEVER.^31^

### Study population and sampling

The study population comprised women who had recently given birth in any of the 10 MOUs and returned for a postnatal follow-up visit at primary healthcare facilities from 3 days to 6 weeks after delivery. Contextual constraints prevented sample realisation through random sampling, and a convenience sample of women visiting postnatal care clinics was obtained.

The sampling design was based on historical data of annual deliveries at each MOU in 2015 (delivery range: 390–1502). Although a sample size of 800 was planned for both pre- and post-surveys, 653 questionnaires were returned for the baseline assessment and 679 for the end-line assessment. The sampling design was originally based on 90% power for a proportional difference; the observed power was 80% – 85% based on the realised sample.

### Data collection

The baseline and end-line surveys were conducted for both the intervention and non-intervention MOUs using a self-administered survey tool. Trained research assistants approached potential participants to complete the questionnaire anonymously. Participants were asked about their experiences regarding communication, labour, clinical care and respectful care during confinement. Seven items in the questionnaire elicited demographic data. The remaining 25 items were derived from similar validated surveys that focused on the clinical care received and on client satisfaction. More details on the survey tool have been published in a previous study.^32^

The baseline survey was conducted in the period February 2016 to April 2016. After the intensive 3-month engagement with the intervention MOUs between May 2016 and August 2016, the survey was repeated between October 2016 and December 2016. One unit had to be re-sampled between February 2017 and March 2017 because of compromised data.

### Data analysis

**FIGURE 1:**
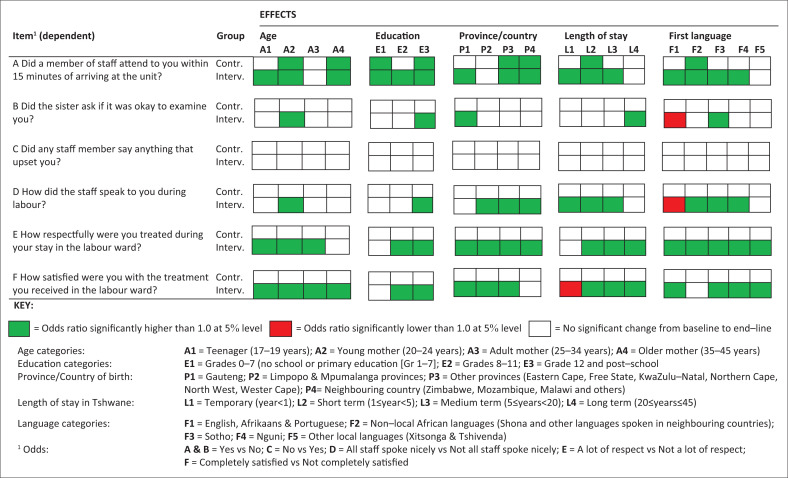
Results of weighted logistic regression modelling of cohorts comparing odds ratios of end-line versus baseline for control and intervention groups (*n* = 1332).

Data were captured on Excel and exported to SAS version 9.4.^33^ Hotdeck imputation^34^ was used to replace missing values in categorical data. Weighting of individual respondents was performed by using the average number of births per month per MOU as the basis, which was obtained from the historical records. The average number of births was then adapted to represent a 2-month period. In the recalculation process, provision was made for the number of births that were recorded over a different number of days per MOU (although, generally speaking, during the same period), separately for the MOUs at baseline and end-line. Finally, weighted percentages were calculated to illustrate the relative representation of each sociodemographic category of the population and each response option. Weighted percentages were also calculated for the responses to individual questionnaire items. Six items covering the main areas of respectful care^14,19^ were selected for analysis in greater depth (see Tables 2 and 3 and Figure 1). Three items (A, B, C) required a Yes/Unsure/No response, and three items (D, E, F) were measured on a four-point semantic scale. The responses to the last three items were dichotomised between the most positive response versus the rest of the responses reduced to a single category.

The results of the six items were analysed using weighted logistic regressions applied to the survey data stratified by MOU and period (baseline/end-line). The analysis was performed using the Procedure Surveylogistic function of the SAS version 9.4 software.^33^ Odds ratios of end-line relative to baseline and also intervention group relative to control group were calculated for each of the six items by applying logistic regression. The average length in days from baseline to end-line per MOU and the number of previous children delivered by each mother were included as covariates in all models. Further inclusions in these models were the effects of period and treatment groups (control/intervention). The logistic models for comparison of each of the effects, such as age, education, province/country, length of stay in Tshwane and first language, were refined by the inclusion of the correction factors ‘average length in days from baseline to end-line’, ‘intervention/control group’ and ‘baseline/end-line’.

### Ethical considerations

The study was approved by the Research Ethics Committee of the Faculty of Health Sciences, University of Pretoria (Protocol 541/2015), and the Tshwane District Research Committee (Project 62/2015). Written permission for the study was obtained from the facility managers of all participating MOUs. No maternal participants could be identified because the survey was completed anonymously. The cover letter of the questionnaire indicated that giving back the questionnaire to the research team was considered informed consent that the information may be used.

## Results

Table 1 illustrates the sample distribution of the two surveys, based on the sociodemographic characteristics of the baseline and end-line respondents. Although these characteristics cannot be controlled in a before-after study with different respondents in the two surveys, the weighting of the statistical modelling made provision for the population characteristics of historically observed births. Because of the small number of participants, English, Afrikaans and Portuguese first-language speakers were grouped as speakers of languages with a European linguistic structure. The age ranges were similar in the two cohorts.

**TABLE 1:** Sociodemographic characteristics of respondents at baseline (*n* = 653) and end-line (*n* = 679).

Indicator	Category	Baseline				End-line			
		Control		Intervention		Control		Intervention	
		*n*†	%‡	*n*	%	*n*	%	*n*	%
Age range (years)	Teenagers: 17–19	12	4.8	38	9.6	27	11.4	34	6.9
	Young mothers: 20–24	78	29.6	123	33.8	80	31.6	133	31.8
	Adult mothers: 25–34	139	53.8	197	47.8	131	52.5	223	53.3
	Older mothers: 35 and above	32	11.8	34	8.9	11	4.5	40	7.9
Province/country of birth	Gauteng Province	117	41.3	198	46.5	113	40.2	257	59.6
	Limpopo and Mpumalanga provinces	66	26.6	82	23.0	38	14.3	60	13.7
	Other provinces (Eastern Cape, Free State, KwaZulu-Natal, North West, Western Cape)	20	7.6	29	7.6	19	7.9	49	10.5
	Neighbouring countries (Zimbabwe, Mozambique, Malawi and other)	58	24.5	83	22.9	79	37.6	64	16.2
Living in Tshwane (years)	Temporary: 0 to < 1	23	9.6	34	10.2	34	15.7	4	1.0
	Short term: ≥ 1 to < 5	60	25.8	66	18.6	62	28.9	59	13.5
	Medium term: ≥ 5 to < 20	87	34.3	120	32.3	62	23.0	122	27.2
	Long term: ≥ 20 to ≤ 45	91	30.3	172	38.8	91	32.4	245	58.3
Languages	Sotho (predominantly Setswana)	102	36.7	166	41.6	85	30.3	218	48.6
	Nguni (isiZulu, isiNdebele, isiXhosa, Seswati)	54	24.4	57	14.7	45	16.3	78	19.6
	Other local languages (Xitsonga, Tshivenda)	21	7.1	86	20.7	18	7.0	70	15.6
	Non-local African languages	58	24.5	80	22.2	78	37.3	63	15.9
	English, Afrikaans, Portuguese	26	7.4	3	0.7	23	9.1	1	0.3
School education	No school or primary education (Grades 0–7)	26	6.6	26	6.6	22	10.3	27	7.2
	Grades 8–11	87	34.3	159	40.5	130	54.1	180	41.6
	Grade 12 and higher education	148	54.6	207	52.9	97	35.6	223	51.2

† , Frequency;

‡ , Weighted percentage.

One observable difference in the end-line survey was the higher percentage of intervention group respondents born in Gauteng (baseline, 47%; end-line, 60%) and the lower percentage born in neighbouring countries (baseline, 23%; end-line, 16%). For the control units, the proportion of respondents born in Gauteng varied by 1% between baseline and end-line; however, there was an increase in the number of respondents born in neighbouring countries (baseline, 25%; end-line, 38%). In the end-line survey, the control and intervention units saw a decline of 12% in respondents born in Mpumalanga and 9% in those born in Limpopo. All the above observations explain some of the differences observed in the length of stay in Tshwane and the first-language mix of respondents between baseline and end-line. Of further note is the higher percentage of respondents with an education level of Grades 8–11 from the control units at the end-line (20% increase) and the lower percentage of respondents with Grade 12 and above (19% decrease). This observation could possibly be explained by the larger representation of foreign respondents in the control group in the end-line survey.

Table 2 focuses on the percentage change in the results of the six items relevant to clinical respectful care that were selected for in-depth analysis. Differences were calculated (1) for baseline and end-line within the control and intervention groups and (2) between the control and intervention groups at baseline and end-line. In the baseline survey, the positive responses for Items A and C started at more or less the same level for the control and intervention MOUs. With regard to women who were attended to within 15 min of arrival (Item A), the control group showed an increase of 15% in positive responses from baseline to end-line and the intervention group, an increase of 21%. For both the control and intervention groups, relatively few respondents reported that remarks they perceived as upsetting had been made to them (Item C). The positive responses of the intervention units for the remaining four items started from a lower level of 14 to 20 percentage points compared to the control units. In the end-line survey, positive responses for the intervention units ‘caught up’ with those of the control units, with minor differences between the two groups’ responses.

**TABLE 2:** Response percentages at baseline and end-line for selected items.

Item Description	Response†	Group	Baseline percentage (%)‡	End-line percentage (%)‡	Percentage point difference§
A. Did a member of staff attend to you within 15 min of arriving at the unit?	Yes	Control	76.6	91.8	15.2
		Intervention	72.6	93.8	21.2
		Percentage point difference¶	4.0	−2.0	-
B. Did the sister ask if it was okay to examine you?	Yes	Control	56.9	61.0	4.1
		Intervention	38.1	64.0	25.9
		Percentage point difference	18.8	−3.0	-
C. Did any staff member say anything that upset you?	No	Control	83.2	89.0	5.8
		Intervention	78.1	91.0	12.9
		Percentage point difference	5.1	−2.0	-
D. How did the staff speak to you during labour?	All staff spoke nicely	Control	61.5	81.0	19.5
		Intervention	47.8	82.6	34.8
		Percentage point difference	13.7	−1.6	-
E. How respectfully do you think the sisters treated you during your stay in the labour ward?	A lot of respect	Control	58.3	71.6	13.3
		Intervention	38.1	74.5	36.4
		Percentage point difference	20.2	−2.9	-
F. How satisfied were you with the treatment you received in the labour ward?	Completely satisfied	Control	63.2	71.1	7.9
		Intervention	47.0	73.6	26.6
		Percentage point difference	16.2	−2.5	-

† , Modelling of responses: Item A: Yes versus No; Item B: Yes versus No; Item C: No versus Yes; Item D: All staff spoke nicely versus Not all staff spoke nicely; Item E: A lot of respect versus Not a lot of respect; Item F: Completely satisfied versus Not completely satisfied.

‡ , Weighted percentage.

§ , Percentage point difference, calculated as end-line minus baseline percentage.

¶ , Percentage point difference, calculated as control minus intervention group percentage.

Table 3 contains an extract of the main results of the weighted logistic regression with odds ratios of end-line relative to baseline and intervention group relative to control group for the six selected items. For Item A (attended to within 15 min), there was a significant positive change in women’s experiences from baseline to end-line for both the control and intervention group units (OR = 6.1 and OR = 8.4; *p* = 0.0007 and *p* = 0.0001, respectively). There was, however, no significant change for both groups for Item C (staff said something upsetting). The remaining four items (B, D, E and F) showed a significant positive change from baseline to end-line regarding the intervention group units (ORs ranging between 2.4 and 4.3, with *p* values ≤ 0.0018). There was no significant change in these items for the control group (ORs between 1.0 and 1.8; *p* ≥ 0.0736). When comparing the intervention group with the control group, the same four items (B, D, E and F) displayed odds ratios significantly below 1 at baseline (OR from 0.4 to 0.6; *p* < 0.0001). At end-line, the odds ratios for all six items (intervention group relative to control group) were above 1.0 (*p* ≥ 0.1434), and all were larger than the odds ratios at baseline.

**TABLE 3:** Results of weighted logistic regression with odds ratios of end-line relative to baseline and intervention relative to control group for selected items.

Item	Response†	End-line relative to baseline			Intervention group relative to control group		
		Group	OR	*p*‡	Period	OR	*p*‡
A. Did a member of staff attend to you within 15 min of arriving at the unit?	Yes	Control	6.098	0.0007***	Baseline	1.204	0.2775
		Intervention	8.418	0.0001***	End-line	1.662	0.1434
B. Did the sister ask if it was okay to examine you?	Yes	Control	0.987	0.9599	Baseline	0.462	< 0.0001***
		Intervention	2.381	0.0018***	End-line	1.114	0.6748
C. Did any staff member say anything that upset you?	No	Control	0.904	0.9725	Baseline	0.838	0.7136
		Intervention	1.215	0.8760	End-line	1.127	0.8493
D. How did the staff speak to you during labour?	All staff spoke nicely	Control	1.801	0.1530	Baseline	0.568	< 0.0001***
		Intervention	3.172	0.0009***	End-line	1.000	0.9990
E. How respectfully do you think the sisters treated you during your stay in the labour ward?	A lot of respect	Control	1.690	0.1079	Baseline	0.443	< 0.0001***
		Intervention	4.334	< 0.0001***	End-line	1.137	0.3602
F. How satisfied were you with the treatment you received in the labour ward?	Completely satisfied	Control	1.763	0.0736	Baseline	0.525	< 0.0001***
		Intervention	4.044	< 0.0001***	End-line	1.204	0.1868

*** , Significant at 1% level.

† , Modelling of responses: Item A: Yes versus No; Item B: Yes versus No; Item C: No versus Yes; Item D: All staff spoke nicely versus Not all staff spoke nicely; Item E: A lot of respect versus Not a lot of respect; Item F: Completely satisfied versus Not completely satisfied.

‡ , Adjusted *p* value = Exceedance probability for accepting H_0_: OR = 1 of ad hoc tests (end-line relative to baseline) and (intervention relative to control).

Differences in odds ratios between baseline and end-line responses are visually compared for the control and intervention groups in Figure 1. The results of the weighted logistic regression modelling of cohorts are broken down by the sociodemographic domains of age, education, province or country of birth, length of stay in Tshwane and first language. Item A (attended to within 15 min) shows odds ratios significantly higher than 1.0 from baseline to end-line for many of the categories across the different sociodemographic domains in both the control and intervention groups, with about twice as many significant domains for the intervention group. Items E (respectful treatment) and F (satisfaction) have significantly higher odds ratios for the majority of categories across the sociodemographic domains in the intervention group only. One category (length of stay in Tshwane < 1 year) under Item F has an odds ratio significantly lower than 1.0, which could possibly be explained by the low number of respondents in this category in the end-line survey (1%). For Item D (staff spoke nicely), the higher odds ratios for the intervention group are concentrated in the domains for the province or country of birth, length of stay in Tshwane and first language. There is a significantly lower odds ratio for English, Afrikaans and Portuguese first-language speakers (< 1% of respondents in baseline and end-line surveys). Item B (consent to examination) has one category in each sociodemographic domain with a significantly higher odds ratio in the intervention group between baseline and end-line and a lower odds ratio for English, Afrikaans and Portuguese first-language speakers. For Item C (staff made upsetting remarks), there was no significant change in any odds ratios from baseline to end-line in either the control or intervention group.

## Discussion

To our knowledge, the ‘working CLEVER’ study^31^ was one of a few studies^35,36,37,38,39,40^ in LMICs that conducted baseline and end-line surveys to measure the change in respectful care after the implementation of an intervention to improve obstetric care. With regard to five of the six questionnaire items selected in our study as a proxy for respectful care during labour, there was a significant improvement in women’s experience of childbirth in the intervention MOUs, compared with only one item in the control MOUs. Item C related to degrading communication on the part of the staff and there was no significant change in women’s experience from baseline to end-line in either the control or intervention group, probably because of the high percentages of positive responses recorded at baseline, although the odds ratio was larger in the intervention group.

The first questionnaire item (A) relates to the withholding of care. The waiting time before being given attention in all units started at approximately the same service level and ended at almost the same improved level. The similarity between the before-and-after performance of the two groups may reflect the effect of the routine engagement of the district clinical specialist team on the improvement of adherence to clinical guidelines in all health facilities. Compliance with minimal patient waiting time is also addressed in different government documents, such as the ‘Ideal Clinic Framework: Definitions, Components and Checklists’,^41^ the ‘Ideal Clinical Manual’^42^ and the ‘National Core Standards for Health Establishments in South Africa’.^43^ The remaining four items (B, D, E and F) relate to obtaining consent before pelvic examinations, communication behaviour, respectful treatment and overall satisfaction with birthing care. Women delivering in the intervention-group MOUs recorded a significant improvement of 26% – 36% in experience in these areas at the end-line versus 4% – 20% in the control group.

Two African studies^35,40^ that recorded the experiences of women regarding consented care focused on other areas of consent, which does not make a valid comparison with our result possible. The *Staha* study was a comparative community and health-system intervention conducted to reduce disrespect and abuse in Tanzania that investigated non-consented care for surgical procedures in an intervention and a comparison between districts before and after the intervention.^35^ Consent to physical examinations was not included in the measurement, and there was no change in consented care practices after the intervention. The *Heshima* project in Kenya^40^ was a before-and-after intervention study that measured women’s experiences of birthing care in 13 facilities using self-report and third-party observation. After the intervention, a highly significant increase was observed in consented care for pelvic examinations, namely, from 61% to 81%, and women reported an increase in abandonment, although it was not statistically significant. Advocacy and influencing providers’ understanding of how to provide better care could improve consented care, but this will be moderated by hurdles in the work environment.^44^

Communication practices in our study improved significantly after the CLEVER intervention. The *Heshima* study in Kenya^40^ recorded a non-significant decline in verbal and physical abuse but a statistically significant reduction of 7% in the proportion of women who felt humiliated or disrespected. The *Staha* study in Tanzania^35^ used language usage and friendly support during labour as care process indicators for respectful care. After the intervention, there was a highly significant improvement in the prevalence of excellent/very good language used in communications with women and the friendly support offered during labour.

In our study, respectful treatment of birthing women and complete satisfaction with treatment improved significantly in the intervention group from baseline to end-line. Tanzania’s *Staha* project^35^ demonstrated a similar improvement at end-line. The authors postulated that the improvement supported the likelihood that the intervention was responsible for the reduction in disrespect and abuse. Although the *Heshima* study in Kenya^40^ demonstrated an overall decrease amongst four of their six typologies of disrespect and abuse, women’s perceptions of respectful treatment or satisfaction with overall treatment during birthing were not measured.

Another study^37^ reported two interventions in a large public hospital in Dar es Salaam, Tanzania – ‘Open Birth Days’ – to prepare women for labour and a workshop for healthcare providers to increase the knowledge of patient rights. The study did not measure specific domains of disrespect and abuse but rather the attitudes and perceptions of providers and women. With regard to women’s perceptions of respect shown by providers and the quality of care received during delivery, no woman rated these as ‘excellent’ in the baseline assessment. After the intervention, ratings of ‘excellent’ for both these items increased to 22.8%. The rating ‘very good’ for quality of care increased from 2.9% to 40.3%. The number of women who reported that they were ‘very satisfied’ with their delivery experience improved by 66.5%, from baseline (10.0%) to follow-up (76.5%). In our study, improvement in respect was measured on a different scale, but there was an improvement of 36.4% in the number of women who reported that they were treated ‘with a lot of respect’. With regard to an improvement in the level of satisfaction experienced, there was an increase of 26.4% in the number of women who reported being ‘completely satisfied’ with their treatment in the end-line assessment.

Our study results indicate that midwives and managers could be engaged to mitigate disrespect and abuse, whilst flexible, adaptable teams of midwives could improve negative birth experiences.^45^

### Study’s strengths and limitations

A strength of this study is the baseline and end-line surveys on experiences of care during childbirth that were conducted at all MOUs in Tshwane district. The same survey tool was used at end-line to obtain a follow-up view of care experiences after the CLEVER intervention.

Limitations of the study relate to the fact that the MOUs in the study could not be randomised but had to be allocated purposively to minimise contamination,^31^ and the women participating in the two surveys consisted of convenience samples of women returning postnatally to a primary healthcare facility. Although there were differences in the demographic data categories between the two survey cohorts, this was mitigated by weighting the data to emulate the population of births. The study was also limited to a single district, and the findings may therefore not be generalisable to other areas. The validity of the data of one of the birthing sites was compromised, and the survey was repeated for that specific site.

## Conclusion

CLEVER Maternity Care is a complex intervention package, and it is not always possible to link specific outcomes to specific components of the package. In addition to the demonstration of a significant reduction in perinatal morbidity and mortality,^31^ it appears as if the implementation of the package contributed towards a better match between women’s expectations of childbirth and midwives’ realities. The CLEVER package set out to change birthing mothers’ experiences of care by providing the skills and tools needed to form small effective teams of midwives, adhering to high-quality respectful obstetric care. The results indicate that the CLEVER package is a potential strategy to address respectful, quality obstetric care and that the integration of CLEVER Maternity Care into district health systems warrants further investigation in more healthcare facilities.
